# Recurrent abdominopelvic solitary fibrous tumours with Doege–Potter syndrome successfully treated with surgical resection following embolization: a case report

**DOI:** 10.1093/jscr/rjag014

**Published:** 2026-01-28

**Authors:** Yuka Yanagida, Akihiro Cho, Yukiko Niwa, Takeshi Ishita, Toshihiko Mori, Moe Tanemura, Atsushi Oda, Ryota Higuchi, Masaho Ota, Satoshi Katagiri, Tadao Nakazawa

**Affiliations:** Division of Gastroenterological Surgery, Tokyo Women’s Medical University, Yachiyo Medical Center, 477-96 Owadashinden, Yachiyo City, Chiba Prefecture 276-8524, Japan; Division of Gastroenterological Surgery, Tokyo Women’s Medical University, Yachiyo Medical Center, 477-96 Owadashinden, Yachiyo City, Chiba Prefecture 276-8524, Japan; Division of Gastroenterological Surgery, Tokyo Women’s Medical University, Yachiyo Medical Center, 477-96 Owadashinden, Yachiyo City, Chiba Prefecture 276-8524, Japan; Division of Gastroenterological Surgery, Tokyo Women’s Medical University, Yachiyo Medical Center, 477-96 Owadashinden, Yachiyo City, Chiba Prefecture 276-8524, Japan; Division of Gastroenterological Surgery, Tokyo Women’s Medical University, Yachiyo Medical Center, 477-96 Owadashinden, Yachiyo City, Chiba Prefecture 276-8524, Japan; Division of Gastroenterological Surgery, Tokyo Women’s Medical University, Yachiyo Medical Center, 477-96 Owadashinden, Yachiyo City, Chiba Prefecture 276-8524, Japan; Division of Gastroenterological Surgery, Tokyo Women’s Medical University, Yachiyo Medical Center, 477-96 Owadashinden, Yachiyo City, Chiba Prefecture 276-8524, Japan; Division of Gastroenterological Surgery, Tokyo Women’s Medical University, Yachiyo Medical Center, 477-96 Owadashinden, Yachiyo City, Chiba Prefecture 276-8524, Japan; Division of Gastroenterological Surgery, Tokyo Women’s Medical University, Yachiyo Medical Center, 477-96 Owadashinden, Yachiyo City, Chiba Prefecture 276-8524, Japan; Division of Gastroenterological Surgery, Tokyo Women’s Medical University, Yachiyo Medical Center, 477-96 Owadashinden, Yachiyo City, Chiba Prefecture 276-8524, Japan; Division of Pathology, Tokyo Women’s Medical University, Yachiyo Medical Center, 477-96 Owadashinden, Yachiyo City, Chiba Prefecture 276-8524, Japan

**Keywords:** solitary fibrous tumour, preoperative embolization, Doege–Potter syndrome, insulin-like growth factor-ii

## Abstract

Solitary fibrous tumours can cause non-islet cell tumour-induced hypoglycaemia, a paraneoplastic syndrome resulting from extrapancreatic tumours secreting insulin-like growth factor -II. This is also known as Doege–Potter syndrome. A male patient in his 70s presented to our hospital with loss of consciousness because of a second relapse of the solitary fibrous tumour with Doege–Potter syndrome. A third surgery was performed after transcatheter arterial embolization. After surgery, blood glucose levels stabilized. Repeated relapses can occur in solitary fibrous tumours even after the complete resection. Embolization of the feeding arteries before resection may be effective in avoiding massive haemorrhage and reducing complications.

## Introduction

Solitary fibrous tumours (SFT) are rare mesenchymal tumours that primarily develop in the thoracic cavity; however, ~30% of SFTs occur in the abdominopelvic region [1]. Most SFTs appear to be benign; however, up to 20% of these tumours have the potential to recur or metastasize [2, 3]. This tumour rarely develops into Doege–Potter syndrome, a paraneoplastic syndrome caused by extrapancreatic tumours secreting insulin-like growth factor-II (IGF-II) resulting in non-islet cell tumour-induced hypoglycaemia [4]. Herein, we present a case of a second recurrence of abdominopelvic SFT with Doege–Potter syndrome that was successfully treated with transcatheter arterial embolization (TAE) and subsequent surgical resection.

## Case presentation

**Figure 1:**
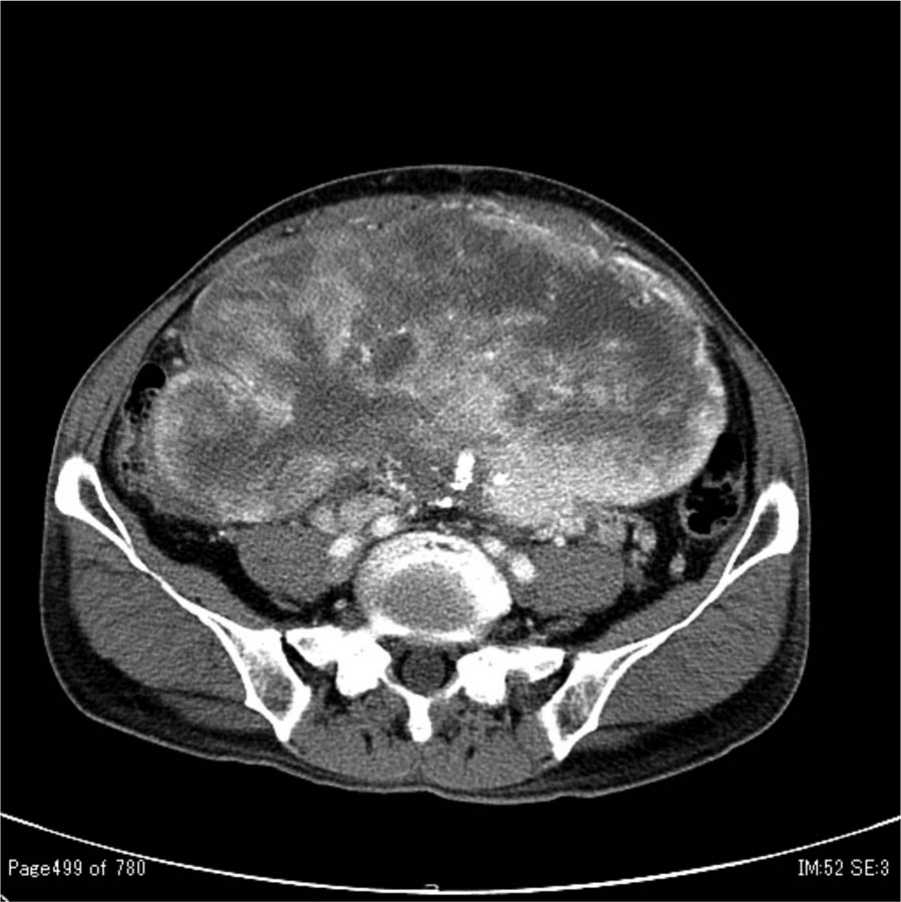
Contrast-enhanced CT showing a large tumour measuring 20 cm in diameter occupying both the abdominal and pelvic cavities.

**Figure 2:**
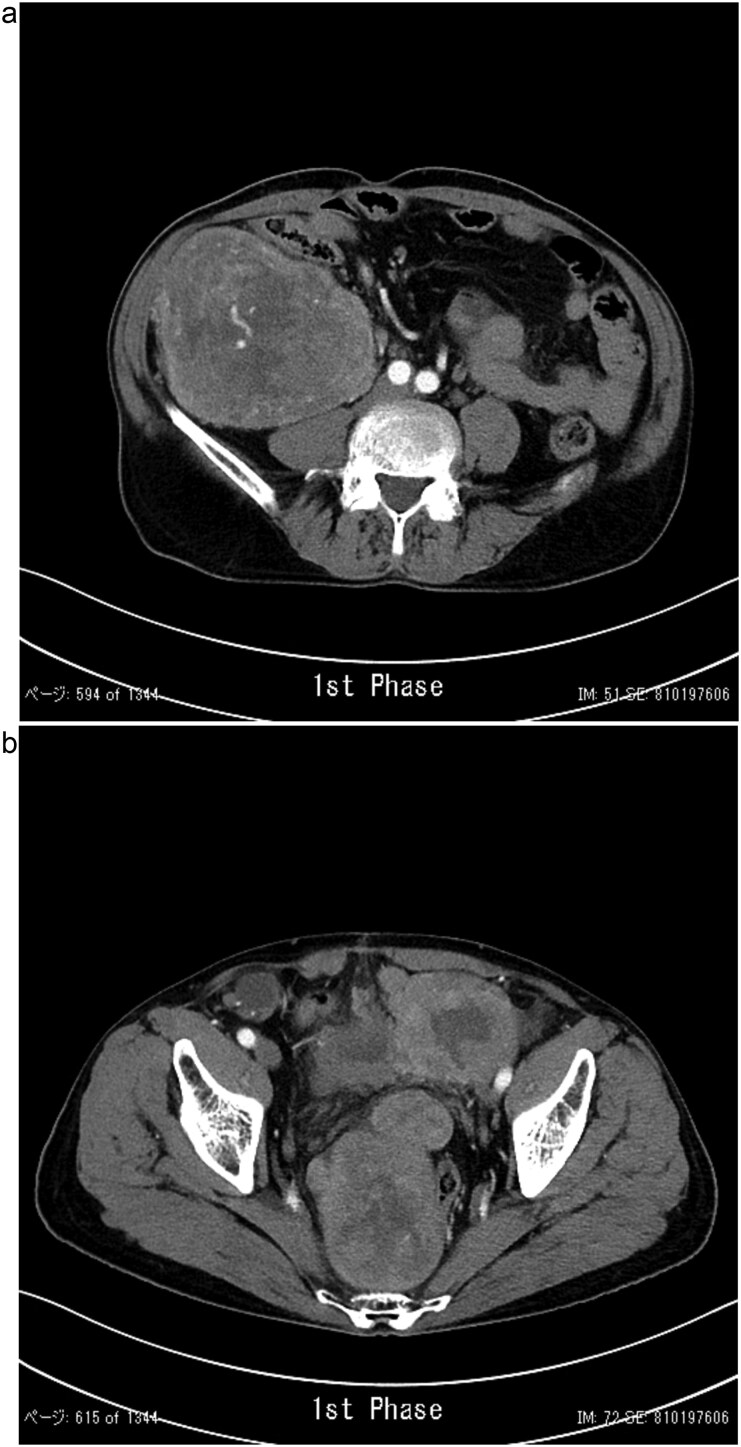
Contrast-enhanced CT showing a lobulated, ill-defined mass with heterogeneous moderate enhancement in the right lower abdomen (a) and several masses occupying the pelvic cavity (b).

**Figure 3:**
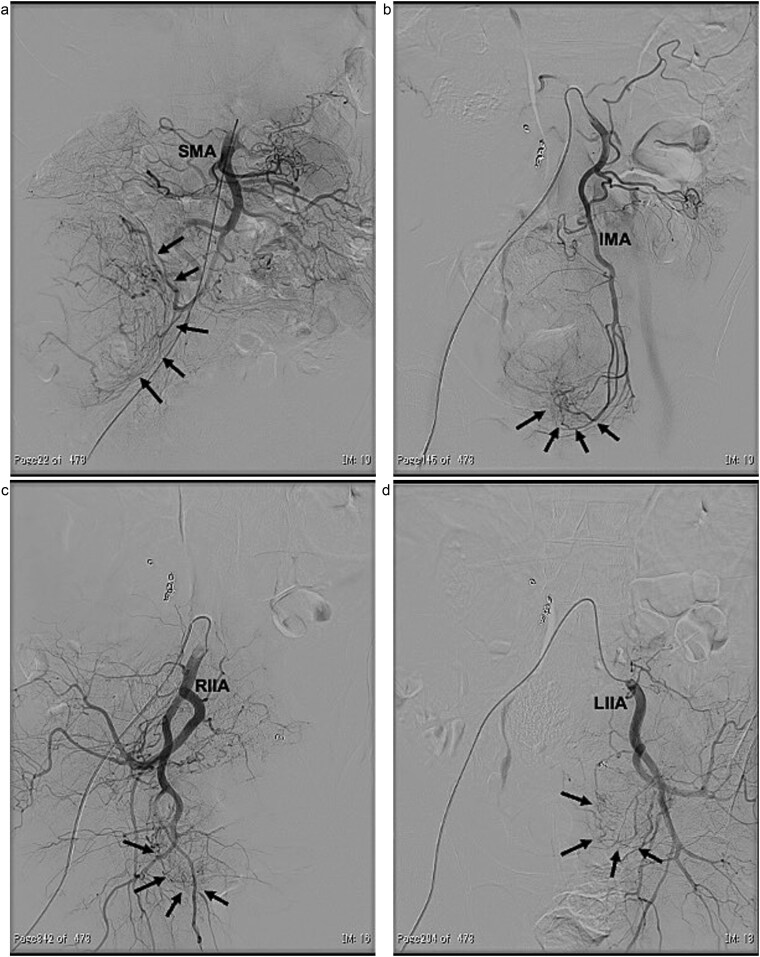
Angiographic findings. The large tumour (arrows) located in the right lower abdomen was supplied by a branch arising from the superior mesenteric artery (SMA) (a). The pelvic tumours (arrows) were supplied by branches arising from both the inferior mesenteric artery (IMA) (b) and the bilateral internal iliac arteries (RIIA and LIIA) (c and d).

**Figure 4:**
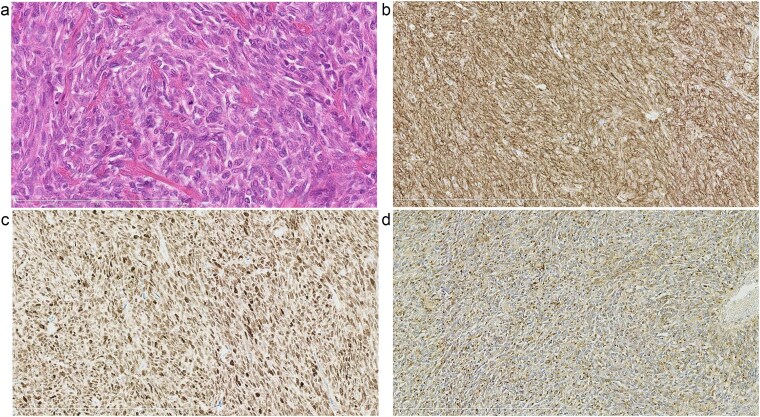
Pathological findings. Haematoxylin and eosin staining of the tumour showing spindle cells exhibiting a ‘patternless’ arrangement in a collagenous matrix (a). Immunohistochemical staining demonstrating that the tumour cells were positive for CD34 (b) and STAT6 (c), consistent with the diagnosis of a solitary fibrous tumour. Immunohistochemical staining showing positivity for insulin-like growth factor -II (IGF-II) in the tumour cells (d).

In 2011, a male patient in his 60s presented to the emergency department with loss of consciousness. His blood glucose level was 20 mg/dL. Contrast-enhanced computed tomography (CT) revealed a large tumour measuring 20 cm in diameter (Fig. 1). Primary surgery was performed and the tumour was diagnosed as an SFT histopathologically. Five years later, contrast-enhanced CT revealed a 4-cm tumour in the lower abdomen. His blood glucose levels were normal. A second surgery was performed; the tumour was excised, and several disseminated nodules were cauterized. Histology revealed that the tumour was an SFT. Eight years later, the patient presented with loss of consciousness. Laboratory data revealed a blood glucose level of 28 mg/dL. Contrast-enhanced CT revealed multiple tumours measuring 3–14 cm in diameter, spread within the abdominopelvic regions (Fig. 2). To prevent excessive intraoperative haemorrhage, preoperative TAE was performed on two larger tumours. Angiography revealed that the large tumour in the right lower abdomen was supplied by a branch of the superior mesenteric artery, whereas the pelvic tumour was supplied by branches from the inferior mesenteric and bilateral internal iliac arteries (Fig. 3). Super-selective catheterization and embolization of the vessels were performed. One week after embolization, a third surgery was performed. Nine tumours were resected. The operative time was 177 min, and the estimated blood loss was 620 mL. Histologically, part of the rectal wall showed necrosis. The tumour comprised spindle cells exhibiting a ‘patternless’ arrangement in a collagenous matrix (Fig. 4a). Immunohistochemical staining was positive for CD34, CD99, Bcl-2, and STAT6, which confirmed the diagnosis of recurrent SFT (Fig. 4b and c). The Ki-67 labelling rate was ~10% in the highly labelled regions. The tumour was also positive for insulin-like growth factor-II (Fig. 4d). After surgery, blood glucose levels completely stabilized. The patient developed a paralytic ileus that resolved spontaneously and was discharged 13 days after surgery. The patient was well, with no recurrence noted during the 10-month follow-up period.

### Discussion

Approximately 4%–11.5% of patients with SFTs develop Doege-Potter syndrome, and hypoglycaemia occasionally is the initial indicator that leads to the detection of large SFTs [5, 6]. In this case, hypoglycaemia was consistent with the initial presentation and the second recurrence. This could be considered an important diagnostic tool for the clinical diagnosis of SFT, both at initial presentation and at disease recurrence [7]. However, hypoglycaemia was not detected during the second relapse, suggesting that it may only occur in larger tumours. The time between the first and second recurrences was shorter than between the initial diagnosis and first recurrence in frequently-recurring SFTs [8]; however, the time between the first and second recurrences was longer in our patient, possibly because the first recurrence was detected using CT before symptoms appeared. Therefore, follow-up diagnostic imaging is crucial for early detection. Surgical resection of large SFTs can be risky and is associated with massive intraoperative haemorrhage of up to 13 660 mL [9–11]. In large pelvic tumours, preoperative TAE enables safe and complete resection of SFTs [12, 13]. We performed a literature review using PubMed with the key words ‘solitary fibrous tumour/SFT’ and ‘embolisation.’ In addition, we manually searched the reference lists of all identified articles to further select relevant articles, and only extracted cases of SFTs occurring in the pelvis. Preoperative TAE with subsequent resection has been previously reported in seven patients (Table 1). Including the present case, there were four women and four men, with a mean age of 56.8 (34–79) years [12–18]. The tumour sizes were 11–30 cm (mean, 12.3 cm). Only two patients presented with hypoglycaemia that improved after embolization. Embolised arteries, including the obturator, internal pudendal, superior vesical, and medial rectal arteries, are predominantly located in the internal iliac artery system. Rectal ischaemia was observed in two of the three cases following embolization of the superior rectal artery. The estimated blood loss was 200–620 mL (mean, 369 mL), which is relatively small compared with previously reported cases [9–11]. Overall, we consider preoperative embolization to be effective for subsequent elective surgery in patients with severe neoplasm bleeding [19, 20]. However, care should be taken to prevent rectal ischaemia, especially for tumours in which the inferior mesenteric artery is the main feeder, even when performing super-selective TAE of the tumour [12]. Considering the potential for intestinal ischaemia, TAE should be performed only a few days before surgery.

**Table 1:** Case reports of preoperative TAE followed by subsequent resection of SFTs occurring in the pelvis.

Author	Age/sex	Hypoglycaemia	Tumour size (cm)	Site of TAE	BS level after TAE	Duration between TAE & surgery (days)	Therapy	Blood loss (mL)	Complications related to TAE
Torigoe [14]	55/F	no	12	RIIA	NA	1	Tumour excision with Hartmann	ND	none
Boe [15]	52/M	no	20	ND	NA	ND	Tumour excision	ND	none
Fard-Aghaie [16]	70/F	no	19	SRA, MRA	NA	14	Tumour excision	200	none
Yokoyama [13]	63/M	no	30	LOA, LIPA, LSVA	NA	30 and 1	Tumour excision	440	none
Yuza [12]	46/F	yes	17	IMA, IIA	stable	2	Tumour excision with LAR	335	rectal ischemia
Yan [17]	55/M	no	12	IIA	NA	ND	Tumour excision	ND	none
Takahashi [18]	34/F	no	11	ROA, RIPA	NA	1	Tumour excision	250	none
Present case	79/M	yes	14	IMA, IIA	stable	7	Tumour excision with LAR	620	rectal ischemia

BS, blood sugar; IIA, internal iliac artery; IMA, inferior mesenteric artery; LAR, low anterior resection; LIPA, left internal pudendal artery; LOA, left obturator artery; LSVA, left superior vesical artery; MRA, medial rectal artery; NA, not applicable; ND, not detailed; RIIA, right internal iliac artery; RIPA, right internal pudendal artery; ROA, right obturator artery; SRA, superior rectal artery.

## Conclusion

Despite our limited experience and the need for future studies to establish the appropriate indications, embolization of the feeding arteries before resection appears to improve surgical outcomes and reduce complications. Given the potential for malignancy, disease recurrence, and metastasis, long-term surveillance using diagnostic imaging is required.
